# Can We Achieve Intuitive Prosthetic Elbow Control Based on Healthy Upper Limb Motor Strategies?

**DOI:** 10.3389/fnbot.2018.00001

**Published:** 2018-02-02

**Authors:** Manelle Merad, Étienne de Montalivet, Amélie Touillet, Noël Martinet, Agnès Roby-Brami, Nathanaël Jarrassé

**Affiliations:** ^1^Agathe Group, Institut des Systèmes Intelligents et de Robotique, UPMC Univ Paris 06, Sorbonne Universités, Paris, France; ^2^Centre National de la Recherche Scientifique, UMR 7222, Paris, France; ^3^Institut National de la Santé et de la Recherche Médicale, U1150, Paris, France; ^4^Centre Louis Pierquin, Institut Régional de Médecine Physique et de Réadaptation, UGECAM Nord-Est, Nancy, France

**Keywords:** upper limb prosthetics, transhumeral amputation, prosthetic elbow control, inter-joint coordination, compensatory strategies

## Abstract

Most transhumeral amputees report that their prosthetic device lacks functionality, citing the control strategy as a major limitation. Indeed, they are required to control several degrees of freedom with muscle groups primarily used for elbow actuation. As a result, most of them choose to have a one-degree-of-freedom myoelectric hand for grasping objects, a myoelectric wrist for pronation/supination, and a body-powered elbow. Unlike healthy upper limb movements, the prosthetic elbow joint angle, adjusted prior to the motion, is not involved in the overall upper limb movements, causing the rest of the body to compensate for the lack of mobility of the prosthesis. A promising solution to improve upper limb prosthesis control exploits the residual limb mobility: like in healthy movements, shoulder and prosthetic elbow motions are coupled using inter-joint coordination models. The present study aims to test this approach. A transhumeral amputated individual used a prosthesis with a residual limb motion-driven elbow to point at targets. The prosthetic elbow motion was derived from IMU-based shoulder measurements and a generic model of inter-joint coordinations built from healthy individuals data. For comparison, the participant also performed the task while the prosthetic elbow was implemented with his own myoelectric control strategy. The results show that although the transhumeral amputated participant achieved the pointing task with a better precision when the elbow was myoelectrically-controlled, he had to develop large compensatory trunk movements. Automatic elbow control reduced trunk displacements, and enabled a more natural body behavior with synchronous shoulder and elbow motions. However, due to socket impairments, the residual limb amplitudes were not as large as those of healthy shoulder movements. Therefore, this work also investigates if a control strategy whereby prosthetic joints are automatized according to healthy individuals' coordination models can lead to an intuitive and natural prosthetic control.

## 1. Introduction

Prosthetic hands have become more and more anthropomorphic in the course of the last decades thanks to the progress in mechatronics, enabling devices to replicate almost perfectly the human hand. For the past years, a myriad of prosthetic hand designs have been proposed and commercialized (Belter et al., 2013), however, fewer solutions have been proposed for the other upper limb joints. Elbow substitution includes passive solutions, like the 12K44 ErgoArm® Hybrid Plus or the 12K50 ErgoArm Electronic Plus® (Ottobock^©^) that can be mechanically- or myoelectrically- locked into a desired position, and active prosthetic elbows, like the DynamicArm 12K100 (Ottobock^©^) or the UtahArm3+ (Motion Control Inc.). The latters, not covered by social security systems in most developed countries, are not affordable for most amputees that are thus fitted with simpler and less expensive prosthetic elbows. Despite the improvement of mechanical features to imitate the human upper limb movements, upper limb amputees, and particularly transhumeral amputees, do not achieve natural movements. In this study, a natural movement refers to a movement that is similar to the body behavior of a healthy individual in terms of shoulder/elbow joint amplitudes, selectivity and synchronicity (Bernstein, 1967). By opposition, amputated individuals equipped with an externally-powered prosthesis perform decomposed upper limb movements, which consist of successive sequences of shoulder, elbow, and wrist movements with large compensatory involvement of the whole body (especially of the trunk), and which require an important cognitive load. Indeed, they often report that current prosthetic devices lack functionality and do not provide the expected assistance in activities of the daily living (ADLs) (Biddiss and Chau, 2007), which leads to the development of compensatory strategies involving the rest of the body, causing shoulder, back, and contralateral limb disorders (Østlie et al., 2011). Subsequently, transhumeral amputees are more likely to reject their prosthesis than transradial amputees (Wright et al., 1995; Biddiss and Chau, 2007).

The counter-intuitive sequential control strategy, along with the device weight and the absence of feedback, is cited as one of the main reasons of limited prosthesis usage (Atkins et al., 1996). Myoelectric control is the most common method to control an externally-powered prosthetic upper limb. Contractions of two antagonistic residual muscles (generally biceps and triceps for transhumeral amputees), measured with surface electromyographic (sEMG) electrodes, are directly controlling a prosthetic function, such as hand opening/closing, or wrist pronation/supination. A combination of muscle contractions, or a co-contraction (i.e. simultaneous contraction of antagonistic muscles), is then required to switch from one mode (e.g., hand closing/opening) to another (e.g., elbow flexion/extension), without being associated with direct prosthetic motion. Although the number of prosthetic joints increases with the amputation level, the same on/off control strategy is applied to forearm and arm prostheses, yielding a dimensionality issue with more controllable degrees of freedom (DoFs) than control inputs. Transhumeral prosthesis users achieve eventually good control of hand and wrist, but have difficulties in general when an active myoelectric elbow is added to the prosthetic arm. Even today, due to sequential and slow prosthetic control, a prosthetic elbow is mostly used for lifting motions and then locked, instead of being involved in global upper limb movements.

Numerous methods like pattern recognition strategies or neural signal interpretation have been developed recently (Castellini et al., 2014) to improve the users possibilities with myoelectric control. However, sEMG signals, often described as unreliable (Bottomley, 1965; Day, 2002), are impeding the implementation of advanced processing techniques. Several studies have investigated alternative control methods to myoelectric signals, such as sonomyography (Sierra González and Castellini, 2013; Akhlaghi et al., 2016), mechanomyography (Silva and Chau, 2003), myokinemetric signals (Abboudi et al., 1999), myokinetic signals (Cho et al., 2016). One possible and yet less explored solution relies on the use of residual limb motion to control a prosthetic limb (Lipschutz et al., 2011; Barton and Sorkin, 2014).

Upper limb prostheses are built with numerous DoFs in order to duplicate the human arm mobility. Hence, like a healthy limb, the prosthesis can perform a movement with an infinite variety of joints configurations. The difficulty is to select the most natural kinematic solution. The current approach of prosthetic devices is based on the association of one neural signal to a unique prosthetic DoF, supposing that the human brain controls each muscle group, and thus each joint, voluntarily and independently. On the contrary, natural limb movements are explained by a coordination between joint kinematics, result of a synchronous control of muscle groups from the central nervous system (Latash et al., 1999). Consequently, healthy movements are task-centered, whereby one focuses on object or hand motion without explicitly controlling each muscle or joint motion. Previous studies have shown evidence of invariant kinematic characteristics in upper limb movements (Roby-Brami et al., 2000; Bockemühl et al., 2010) proving the coordinated aspect of joint movements, and especially of the shoulder/elbow coupling (Lacquaniti and Soechting, 1982; Micera et al., 2005).

Replicating a human-like control strategy whereby joint motion is coupled onto a transhumeral prosthesis is a promising solution. Thus, residual limb mobility, that most transhumeral amputees have, can be used to drive automatically the elbow joint, as originally presented in Gibbons et al. (1987) who developed a mechanical system that links residual limb motion to elbow flexion and wrist rotation. If the inter-joint coordination relationship is known, then distal joint motion (e.g., elbow flexion) could be predicted from measurement of proximal joint kinematics (e.g., shoulder). To this aim, research groups have been focusing on modeling the healthy shoulder/elbow coordination during common gestures like pointing or grasping. Several regression tools have been utilized to approximate the nonlinear function relating shoulder to elbow kinematics, however artificial neural networks (ANNs) seem to give the best prediction results. The study in Kaliki et al. (2008) used an ANN-based architecture to estimate offline distal joint kinematics from recordings of healthy individuals' pointing movements: the selected set of ANN inputs required the measurement of three shoulder angles and two shoulder translations to predict elbow flexion angle and forearm rotation. In Iftime et al. (2005), an upper limb inter-joint coordination model was derived from kinematic data of healthy individuals moving objects placed on a plane surface: a radial basis functions network (RBFN)-based regression was used to approximate the shoulder/elbow relationship. In most previous approaches, the training data sets were recorded with camera-based motion capture systems, which cannot be used easily outside laboratory environments, especially in the prosthesis users environments. It is only recently that the development of accurate embedded motion sensors like inertial measurement units (IMUs) and computing power improvement of micro-controllers have enabled the implementation of an automatic prosthetic control strategy using inter-joint coordination models. Nonetheless, the approaches and models presented in the literature have not yet been tested on prosthetic devices. In Mijovic et al. (2008) and Farokhzadi et al. (2017), elbow flexion could be estimated offline with accelerometer-based shoulder kinematic measurements, yet the control strategy was not implemented. Similarly, the recurrent relationship between humerus elevation (i.e. angle between the humerus longitudinal axis and the trunk vertical axis) and wrist pronation/supination was investigated in Montagnani et al. (2015) with an IMU-based training data set and a principal component analysis (PCA)-based regression method. Most recent results combine IMU-based shoulder kinematics data and residual limb's myoelectric activity to build the inter-joint coordination model. In Akhtar et al. (2012), EMG signals from arm and deltoid muscle groups were added to shoulder angles data as inputs of an ANN-based model: elbow and forearm rotation angles were estimated offline using a training data set recorded with healthy participants. Comparably, a set of coefficients linearly relating the humerus elevation angle and the EMG signals to the elbow angular velocity was found in the study in Alshammary et al. (2016); they were used in real time by healthy individuals to control a virtual prosthesis.

Despite promising offline prediction results, the paradigm whereby the residual limb motion and the motorized elbow are coupled based on inter-joint coordination models has not been tested on a prosthesis since the work of Gibbons et al. (1987). The aim of the present study is to test a similar paradigm with a transhumeral amputee using a prosthesis. Preliminary work was focused on concept validation with healthy subjects controlling a prosthetic forearm implemented with the participants' own model of shoulder/elbow coordinations (Merad et al., 2016a). Like other studies in the literature, the inter-joint coordination model was built using the data from healthy gestures recordings. However, the shoulder/elbow coupling cannot be recorded with a transhumeral amputee. A possible solution, investigated by Merad et al. (2016b), combines the inter-joint coordination data from several healthy individuals to build a generic model. The present study investigates the outcomes of automatizing the elbow motion according to residual limb movements during an experimental session with a transhumeral amputee. The latter pointed at targets with a prosthesis prototype including an motorized elbow implemented with a generic inter-joint coordination model from two healthy persons' kinematic data. For comparison, the participant performed also the task with the prosthesis implemented with his own myoelectric control strategy.

## 2. Materials and methods

A novel control approach whereby the prosthetic elbow motion is automatically-driven by the residual limb motion was tested with a transhumeral amputated individual. The study was divided into two phases, performed several days apart: the training data set acquisition, and the control test. During the first part of the experiment, healthy individuals performed the pointing task while their left upper limb kinematics was recorded. Shoulder and elbow angular velocities were utilized to build a generic model of the left inter-joint coordination that included both subjects pointing strategies. During the control test, a left-amputated transhumeral participant used a prosthesis prototype implemented with the healthy data-based coordination model to point at targets. To further elucidate the outcomes of this automatic control approach, the participant performed the task also with his own myoelectric control strategy replicated on the same prototype.

### 2.1. Participants

Two healthy individuals and one transhumeral amputee participated in the study. This study was carried out in accordance with the recommendations of the Université Paris Descartes ethic committee CERES, which had approved the protocol. All subjects gave written informed consent in accordance with the Declaration of Helsinki. Two right-handed able-bodied individuals (one male, 1.82 m, and one female, 1.72 m, both 25 years old) were recruited for the training data set acquisition experiment.

The transhumeral amputated individual who took part in the experiment was 34 years old (height 1.80 m), and underwent a transhumeral amputation of the left limb in 2014 after a work-related accident. The inclusion criteria were a long residual limb, good residual limb mobility, absence of phantom limb pain, no brachial plexus damage, myoelectric prosthesis user, and a prosthesis socket and harness that allowed some residual limb mobility. The range of motion without socket and harness of the selected participant's residual limb was within the values of a healthy shoulder's range of motion. When wearing the prosthesis, he could do a shoulder flexion of 50°, a shoulder extension of 30°, a shoulder abduction of 40°, but the socket prevented humeral axial rotation. Since the amputation, the participant was equipped with an i-Limb Touch Bionics hand and a motorized wrist rotation. He received a myoelectrically-controlled elbow (UtahArm3+, Motion Control Inc.) a couple of months before being recruited for the experiment. Hence, he was considered to be trained with myoelectric control using biceps and triceps contractions. However, a poor control over triceps contractions, and hence co-contractions, limited the participant's myoelectric capabilities: his myoelectric control strategy, detailed thereafter, had to be adapted to ease his daily prosthetic usage.

### 2.2. Protocol

**Figure 1:**
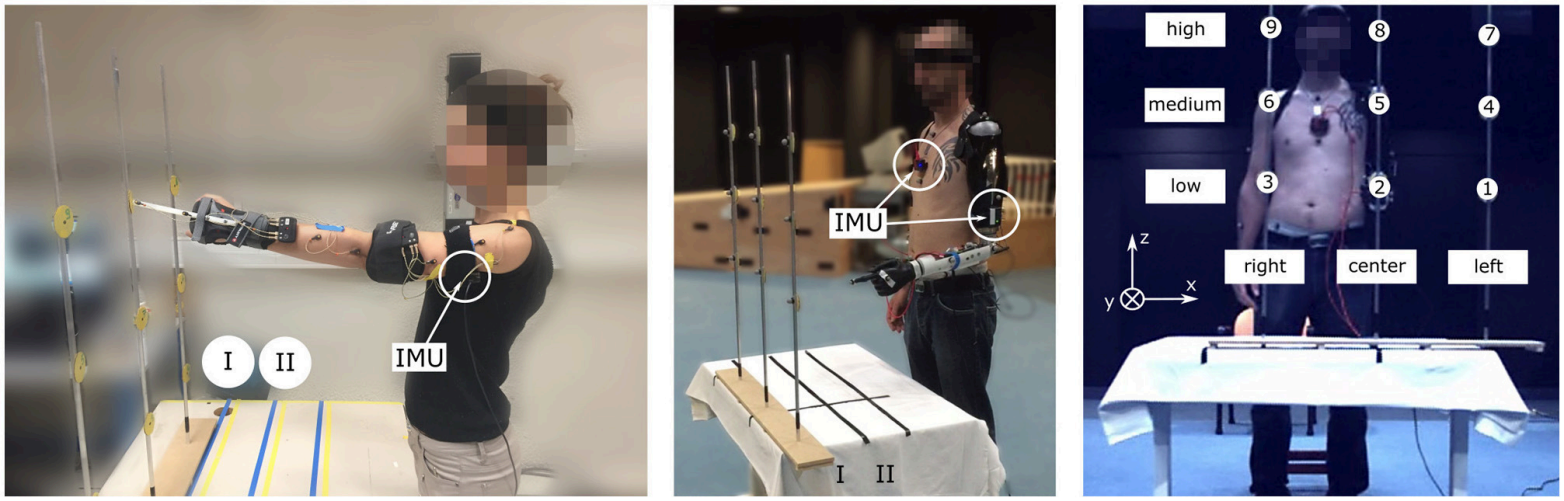
Experimental setup with healthy **(Left)** and amputated **(Middle)** participants. All subjects, equipped with two IMUs (chest and arm) measuring the shoulder kinematics, pointed at 18 targets with the left arm. The targets were distributed such that there were 9 targets at each distance (maximum I, intermediate II) **(Right)**.

The task was the same for all participants; they were instructed to point at targets with their left limb; healthy individuals used a rod attached to a wrist splint's back instead of their index, whereas the amputated individual achieved the task with the prosthetic index. The initial position was defined with the left elbow flexed at 90°, and the wrist rotated such that the thumb was pointing upward, as shown in Figure 1. The prosthetic hand was set in the pointing posture (all fingers except index were flexed) at the beginning of the trial. Even though hand and wrist could be myoelectrically-controlled, the amputated individual was instructed to use only the elbow during the session. The healthy subjects were asked to maintain the same hand orientation during the movement, i.e. to maintain the hand with the thumb up, such that they performed in the same conditions as the amputated participant. For each pointing movements, the subjects stayed immobile in the initial position until told the target number to reach, then brought the finger/rod tip the closest to the target, stayed immobile until instructed to come back to the initial position. No particular instruction was given to the subjects concerning movement duration, speed, or target reaching strategy. Healthy subjects repeated the task twice. The transhumeral amputated participant performed the task once with the prosthetic elbow in myoelectric control mode (ME-mode), and once in automatic mode (A-mode).

### 2.3. Prototype

#### 2.3.1. Hardware

**Figure 2:**
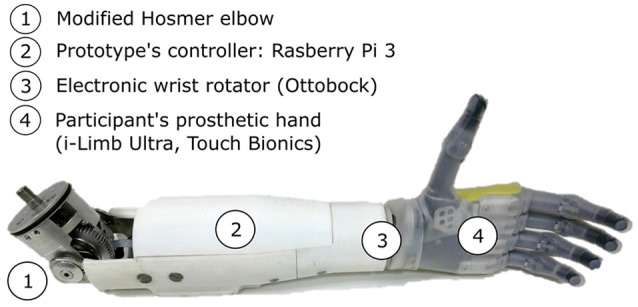
The two-DoF forearm prototype includes a motorized elbow (1) and an electronic wrist rotator (3). The participant's prosthetic hand is connected to the forearm (4). The prosthetic components are controlled by a Raspberry Pi 3 (2) reading the myoelectric signals from the participant's surface electrodes, and from two IMUs.

Commercialized pieces like a conventional electronic wrist rotator (model 10S17, Ottobock^©^), and an E-TWO electric elbow (Hosmer, Fillauer) were assembled to form a two-DoF prosthetic forearm, as depicted in Figure 2. Any myoelectric prosthetic hand with the Quick Disconnect system could be interfaced with the prototype. During the experiment, the amputated individual's i-Limb hand was mounted on the prototype to perform the task. A Raspberry Pi 3^©^ controlled the prosthesis electronics, as well as a motor controller (Ion Motion Control^©^) in charge of elbow's and wrist's motor speed control. An encoder was added to the elbow motor for closed-loop control purpose. The forearm structure, in which most of the electronics was located, had been printed in ABS and reinforced with metal parts. The prosthetic forearm weighed 810 g without a prosthetic hand attached to it. The prosthesis prototype was mounted onto the subject's own socket, and his two myoelectric electrodes (Myobock, Ottobock^©^), located within his prosthesis socket and placed over the residual biceps and triceps groups, were connected to the prototype's controller. The latter, which also read the data from two IMUs (x-IMU, x-io Technologies), piloted the prosthetic joints according to the input signals and the control mode.

#### 2.3.2. Prototype control

Two control laws were implemented on the prototype. The myoelectric mode (ME-mode) control corresponded to the amputated participant's own myoelectric control strategy that was duplicated on the prototype's controller. The selected participant used the following 2-myoelectric-site sequential strategy:

- First, a biceps contraction controlled the elbow flexion until the forearm was positioned. When the contraction stopped, the control switched automatically to the end-effector control.
- Flexion (resp. extension) of hand fingers was controlled by slow biceps (resp. triceps) contractions.
- Wrist pronation (resp. supination) was controlled by fast biceps (resp. triceps) contractions.
- A co-contraction switched back to elbow control, and lead to a rapid and uncontrolled elbow extension.

Therefore, if an elbow extension or flexion was required after setting the elbow angle to the 90-degree initial position, the prosthesis user had to do a co-contraction to unlock the elbow that extended rapidly, then to do a biceps contraction to flex the elbow and reach the desired angle. The prototype's parameters for myoelectric control were copied from his own prosthesis, including the velocities for elbow flexion and extension.

The automatic mode (A-mode) control strategy used a shoulder/elbow coordination model, built from the healthy subjects' pointing movements, to estimate the elbow angular velocity based on IMU-based residual limb's kinematic data. Hence, the shoulder joint drove automatically the elbow flexion/extension movements. Meanwhile, hand and wrist could still be controlled via the myoelectric signals, but the transhumeral amputated participant was instructed not to use these DoFs to achieve the task.

### 2.4. Setup

Healthy participants pointed at targets with the tip of a rod attached to a wrist splint's back, used to prevent wrist flexion during the movements, while the amputated participant wore the prosthesis prototype with his own prosthetic hand plugged in and achieved the pointing task with the prosthetic index's tip. The experimental setup is illustrated in Figure 1. The IMUs were placed on the participants' chest and arm/socket. They were connected via USB first to a laptop that recorded the data in the experimental setup with healthy subjects, then to the prototype's controller during the experimental session with the transhumeral amputee. A camera-based motion capture system, only used for off-line data analysis, recorded the participants' upper body kinematics at a frequency of 100 Hz: a Codamotion system (Charnwood Dynamics, Ltd.) was utilized with the healthy subjects, and a Vicon^©^ system (Vicon Motion System, Ltd.) was used with the amputated participant. The main markers locations for both motion capture systems were: left index's middle phalanx, left hand's back, left forearm, left elbow lateral epicondyle, left upper arm, left and right acromions, suprasternal notch, xiphoid process, left and right anterosuperior iliac spines. In the second experimental setup, two additional video cameras, synchronized with the Vicon's kinematic data, recorded the scene. Moreover, two force plates recorded at a sampling frequency of 1 kHz the force applied by each foot.

The task consisted in pointing at targets, numbered from 1 to 9 and attached to three sticks; they were presented at 2 different distances (I, II), as illustrated in Figure 1. The targets positions were adjusted for each subject depending on the arm length and shoulder height: target 8 was aligned with the subject's left shoulder such that the subject could reach it by extending fully the left arm, as shown in Figure 1. Target 2 was placed below target 8 at the left anterosuperior iliac spine height, and target 5 was placed halfway between target 2 and 8. The distance II corresponded to the distance I (arm length) to which 15 cm were subtracted, as illustrated in Figure 1. The distance between the center and the lateral targets, i.e. between targets 1 and 2, and 2 and 3, was arbitrary fixed to 30 cm for all subjects.

### 2.5. Data processing

#### 2.5.1. Generic model

**Figure 3:**
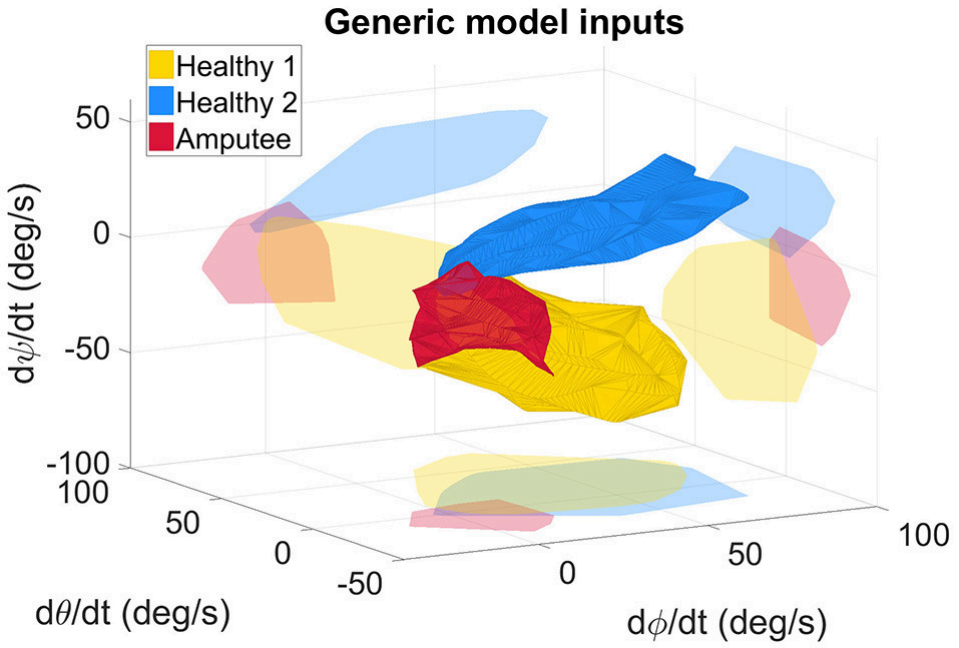
Measured angular velocities, inputs of the generic model, for the healthy and amputated participants. The light-colored forms represent the projection of the solid forms on a plane for better 3D representation. The angles ϕ, θ, and ψ represent the 3 Euler angles. The angular velocities represented on the graph were fed to the RBFN-based regression algorithm either to build the inter-joint coordination model (in the case of healthy subjects' data), or to estimate online the elbow motion with the measured shoulder kinematics (with the amputee's data).

Kinematic data from the two healthy subjects were recorded while they performed the pointing movements. The two IMUs (trunk and arm) provided information on their own orientation with respect to an initial reference frame. The latter was defined during a calibration phase whereby the two sensors were aligned such that they shared the same initial reference frame. The orientation information was represented by a quaternion value, result of each IMU's embedded fusion algorithm (Madgwick, 2010). The rotation matrix was derived from the relative orientation between the two IMUs. The rotation matrix coefficients were then utilized to compute the Euler angles ψ, θ, ϕ (ZYX sequence) which were chosen to describe the arm kinematics with respect to the trunk. The angle β, which represented the elbow flexion angle, was derived from the Codamotion measurements. Shoulder and elbow angular velocities were computed numerically from the shoulder and elbow angles. They were partitioned for each movement (9 targets, 2 distances, 2 trials, i.e. 36 movements), and low-pass filtered with a cutoff frequency of 2 Hz. The shoulder angular velocities are depicted in Figure 3 that shows that the two able-bodied individuals had different pointing strategies.

An inter-joint coordination model, built from the two able-bodied subjects' kinematic information, served as mapping between the shoulder angular velocities and the elbow angular velocity. This model was a combination of the healthy subjects' coordinations, and thus was referred to as generic model. As commonly performed in the literature, an RBFN-based regression method was implemented in a MATLAB script to model the nonlinear relationship between the shoulder and elbow angular velocities; the relationship's analytic form was a linear combination of Gaussian components chosen as the radial functions, as explained by Stulp and Sigaud (2015). A training phase utilized the training data set (measured quadruplets ($\dot{\psi }$, $\dot{\theta }$, $\dot{\phi }$, $\dot{\beta }$) of selected movements) to compute the model's coefficients. The obtained relationship was implemented on the prosthesis controller, and was used to estimate the elbow angular velocity $\dot{\beta }$ from online IMU-based shoulder angular velocities ($\dot{\psi }$, $\dot{\theta }$, $\dot{\phi }$), also calculated with respect to the trunk orientation.

#### 2.5.2. Data analysis

The transhumeral amputated participant performed 18 movements (9 targets, 2 distances) for each control mode (ME-mode, and A-mode). The video recordings, synchronized with the Vicon^©^ data, were utilized to cut the position and force recordings into short data segments, one for each movement toward a target. Since the participant never actuated the prosthetic elbow during the pointing movement itself, but always prior to the movement, the data segment for movements with ME-mode were started after the forearm pre-positioning phase. The data segments were analyzed to compare the participant's body behavior when the task was done with a myoelectrically-driven elbow or with an automatically-driven elbow. The task performance was assessed with the precision error and the task completion time. The precision error was defined as the distance between the target and the end-effector's position when the subject stopped the movement. The movement duration corresponded to the time needed to do the movement without considering the forearm pre-positioning in ME-mode; it was calculated based on the end-effector's velocity norm.

The analysis was also focused on the compensatory strategies developed by the subject to achieve the task. Trunk movements were assessed with the trunk inclination angle, i.e. the angle between the final and initial position of the trunk's main axis. The latter was defined as the line going through the pelvic center (barycenter of the sacrum, right and left anterosuperior iliac spines markers), and the thorax center (in between the C7 and clavicle markers). The trunk displacements were also evaluated with the cumulative path of the thorax center, calculated as the sum of the distances between two consecutive points of the trajectory, and with the hip forward displacements, i.e. the range of motion of the pelvic center in the anteroposterior direction. In addition, changes in the weight distribution during the movements were assessed by computing the difference between the final and initial amounts of force applied by the left foot with respect to the total force applied by both feet. The amplitude of the residual limb motions was evaluated with the humerus elevation angle, i.e. the angle between the humerus longitudinal axis and the trunk main axis, derived from the IMUs measurements. Residual limb movements were compared to the healthy arm movements from the generic model's training data set.

## 3. Results

### 3.1. Functional assessment

**Figure 4:**
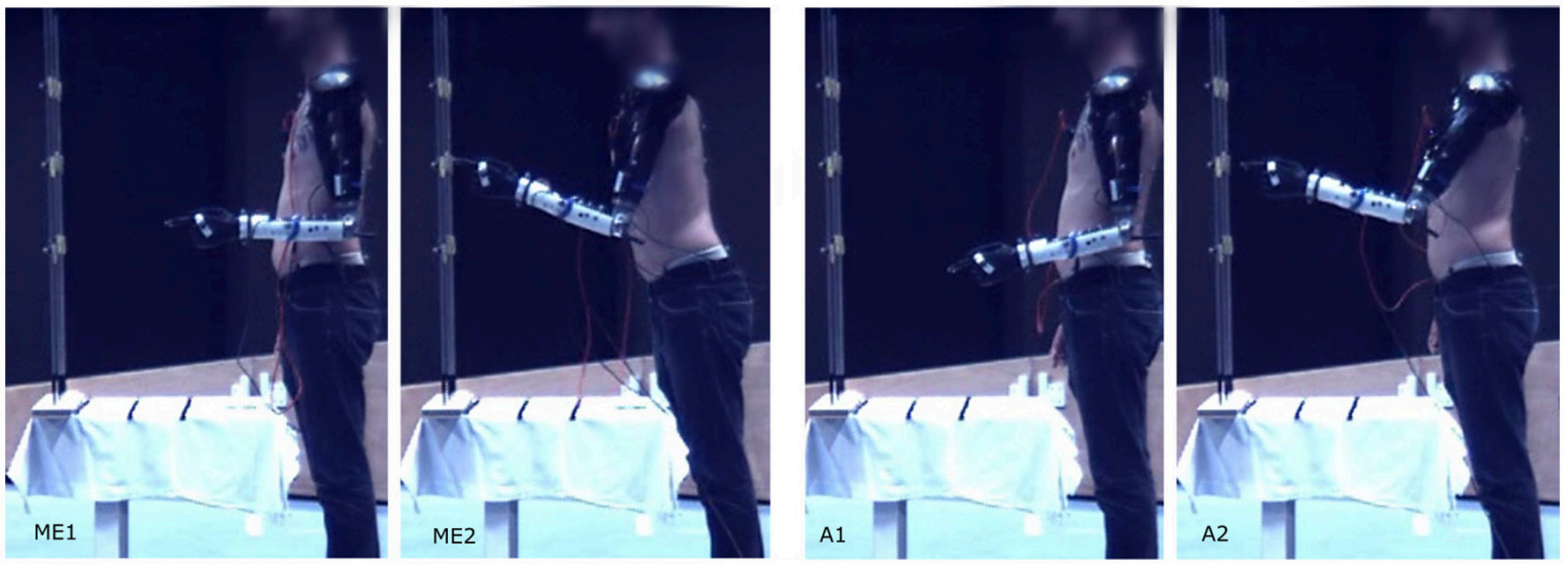
Pointing movement toward target 5 at distance I performed with myoelectric control (ME1-ME2), and automatic control (A1-A2).

**Figure 5:**
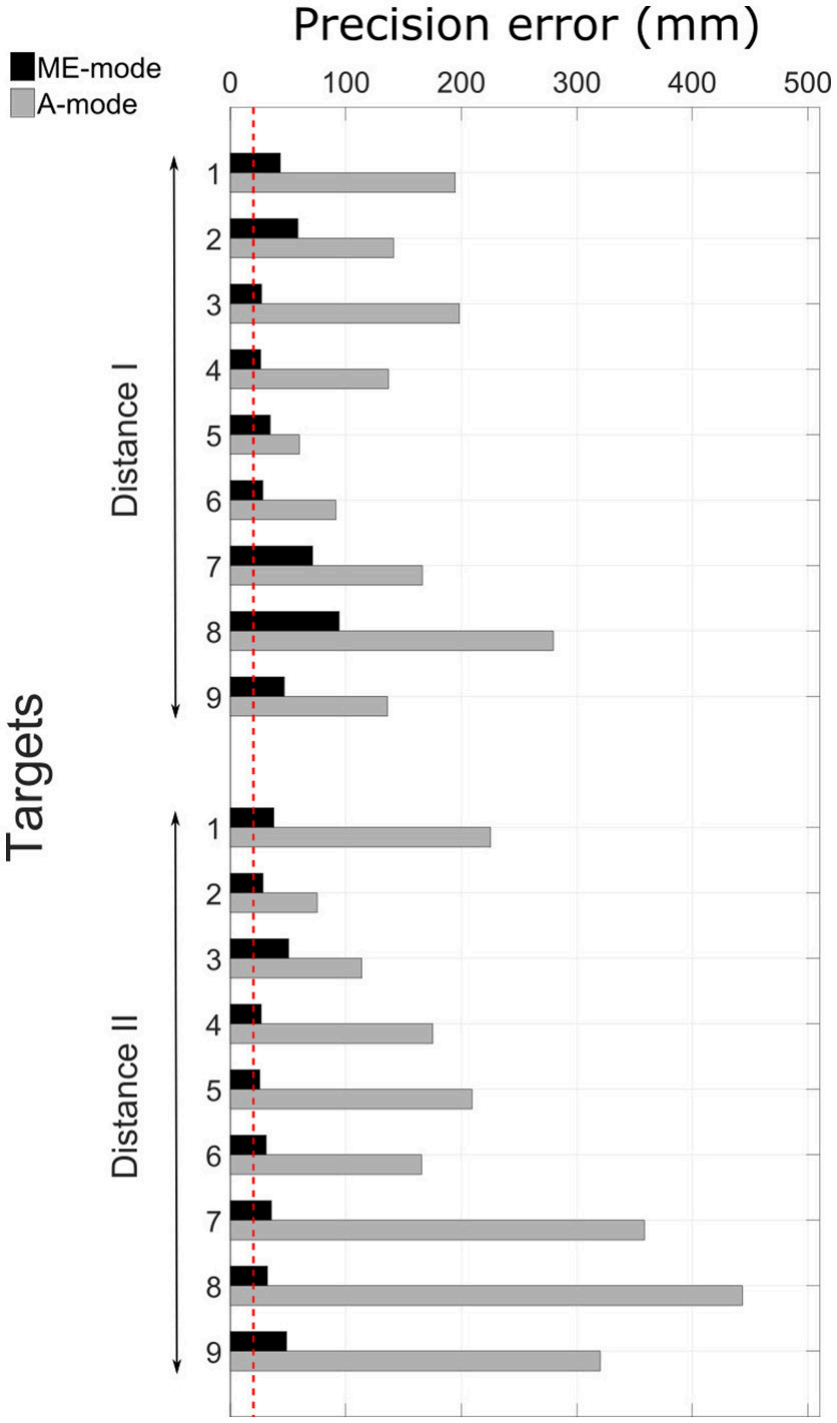
Precision errors in ME-mode and A-mode for all targets. The red dotted line represents the precision error offset of 20 mm that accounts for the finger marker position. The targets distribution can be seen in Figure 1.

A typical pointing movement is illustrated in Figure 4. The pictures represent the initial and final postures of the movement performed with the prosthetic elbow in ME-mode (ME1 and ME2), and in A-mode (A1 and A2). The participant could not reach all the targets with A-mode, as confirmed by the precision error results depicted in Figure 5. The overall error values, averaged over all targets and distances, was 41.5 ± 18.3 mm in ME-mode, and 193.9 ± 101.2 mm in A-mode. To limit marker occlusion, the finger marker was placed on the middle phalanx of the prosthetic index. Hence, there was an offset of 20 mm when the finger touched the target.

The movement durations were similar for the two control modes: the pointing motion lasted 1.82 ± 0.46 s with ME-mode, and 1.92 ± 0.68 s in A-mode. As a comparison, the movements of the two healthy subjects recruited for the generic model data acquisition lasted 1.37 ± 0.30 s in average. However, the calculation did not account for the reconfiguration time needed by the participant to position the prosthetic forearm in ME-mode. As explained in section 2.3.2, the participant did not have control over elbow extension with his own myoelectric control strategy: elbow flexion was controlled by biceps contractions, and the release of passive elbow extension was triggered by a co-contraction. When considering the forearm re-positioning before the actual pointing motion, the movements duration increased by up to 9 s in ME-mode.

### 3.2. Overall movement strategy assessment

**Figure 6:**
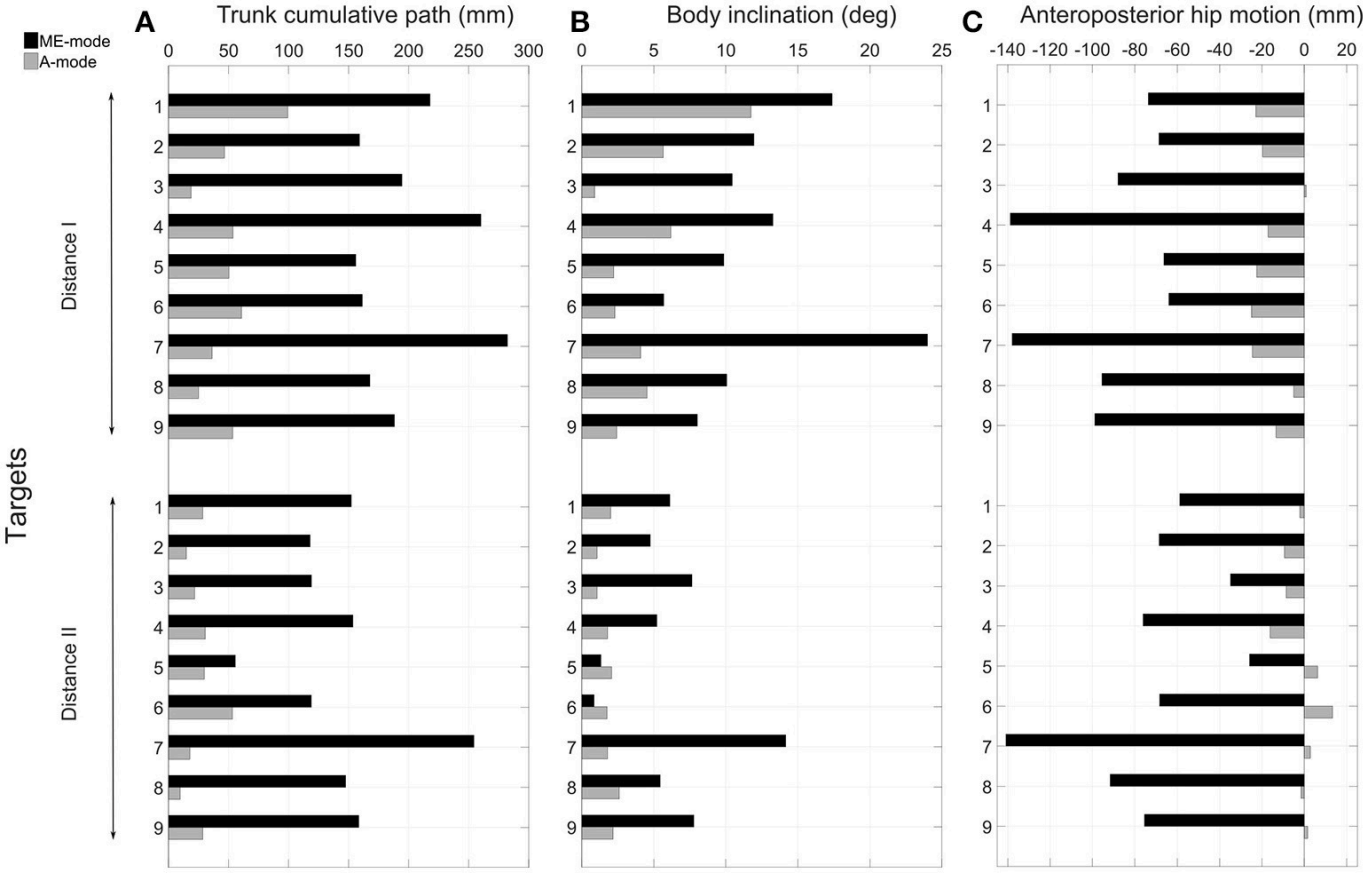
Analysis of compensatory trunk movements. The cumulative trajectory of the thorax center is represented in **(A)** quantifying the trunk's displacements during all movements and for the two conditions of control. The range of motion of the trunk main axis is represented in **(B)**. The hip anteroposterior displacements are depicted in **(C)**; a forward motion is represented by a negative values (see reference frame in Figure 1).

**Figure 7:**
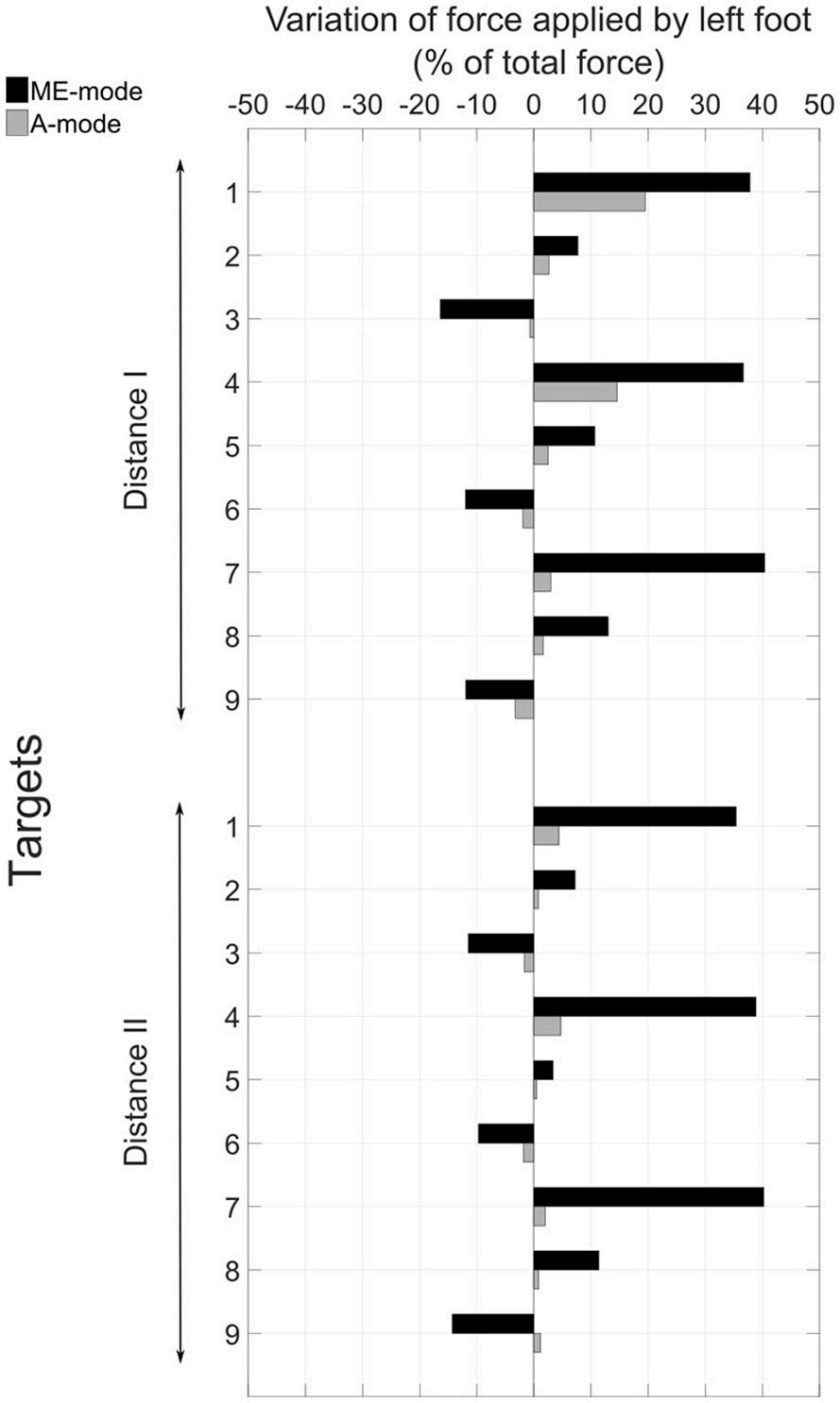
Variation between the beginning and the end of the movement of the amount of force applied by the left foot with respect to the total force.

The control mode of the prosthetic elbow influenced the overall body behavior. Large compensatory movements were observed in ME-mode, and they were reduced when shoulder and elbow motions were coupled (A-mode). Indeed, since end-effector position was mostly adjusted with trunk motions with myoelectric control, body displacements were larger in ME-mode: the thorax center's cumulative trajectory, shown in Figure 6A was 170.2 ± 56.2 mm in ME-mode, and 37.6 ± 21.8 mm in A-mode, averaged over all targets and distances. When the elbow was myoelectrically-controlled, the participant brought the end-effector close to the target by leaning toward the targets, yielding large body inclinations (Figure 6B): the range of motion of the body inclination angle was 9.1 ± 5.7° in ME-mode, and 3.1 ± 2.6° in A-mode, averaged over all distances and targets. The values of the hip displacements in the anteroposterior direction (Figure 6C) also illustrates larger trunk mobility when doing movements with myoelectric control. Behavior modifications between the ME-mode and the A-mode could also be observed with changes in the forces distribution: using an automatically-driven elbow reduced inequalities between the forces applied by the feet. The values in Figure 7 represent the variations of the amount of force exerted by the left foot with respect to the total force. They showed some important differences between the two control conditions: indeed, the participant's weight shifted more toward the left foot (i.e. the amputation side) during pointing movements performed with myoelectric control.

**Figure 8:**
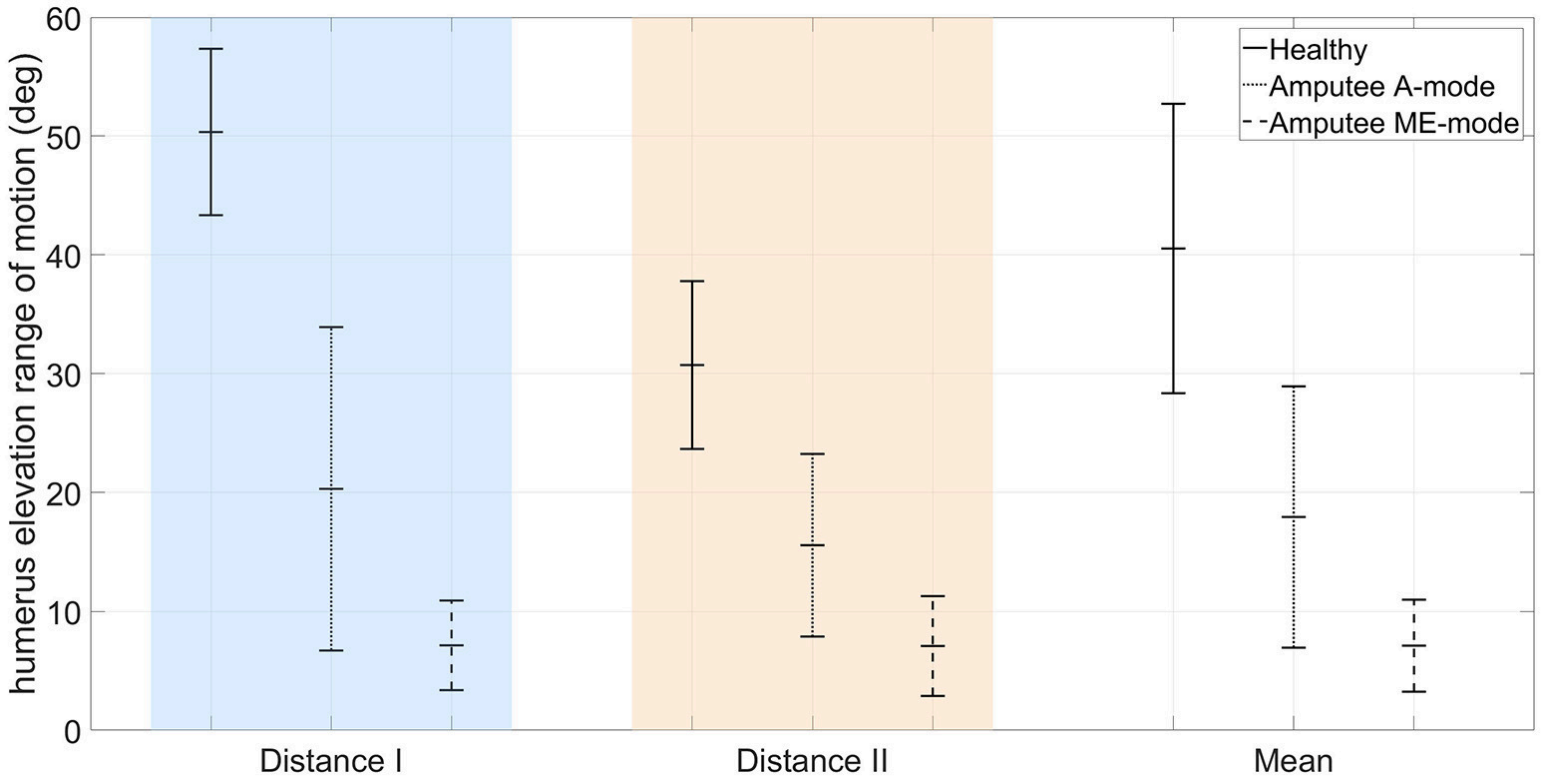
Comparison of arm elevation's range of motion between the mean of two healthy participants, and a transhumeral amputee using a residual limb motion-driven elbow (A-mode), or a myoelectrically-driven elbow (ME-mode).

The residual limb motion was different from one condition to another. The A-mode required the participant to use his residual limb to achieve the task, whereas most movements with myoelectric control were performed with the trunk after setting the prosthetic forearm into the adequate position. Consequently, humerus elevation values were very different from one control condition to the other: averaged values over targets targets and distances were 7.1 ± 3.9° in ME-mode, and 17.9 ± 11.0° in A-mode. For comparison, the pointing movements of the two able-bodied subjects recruited in the experiment's first part were also analyzed. The overall arm elevation values are 40.5 ± 12.6° for the healthy subjects. The Figure 8 depicts the humerus elevation's ranges of motion of the healthy and amputated participants, averaged over the targets of each distance. In addition to the shoulder kinematics of the able-bodied individuals, the transhumeral amputated participant's shoulder angular velocities used as inputs of the inter-joint coordination model are shown in Figure 3.

## 4. Discussion

A transhumeral amputated individual was asked to point at 18 targets split in 2 groups, one for each distance. The subject performed the task with a motorized elbow controlled either by his own myoelectric control strategy (ME-mode), or by an RBFN-based regression model of healthy inter-joint coordinations coupling residual limb motion to prosthetic elbow flexion/extension (A-mode).

### 4.1. Precision error

The task performance assessment showed that the precision error values were larger when the task was performed with an automatically-driven elbow. Even though the participant was selected for his residual limb's mobility, high-located targets were out of reach because he could not lift the residual limb and the prosthesis to the appropriate height. This limitation was due to either the pain exerted by the prosthesis on the residual limb's distal part, or to the prosthesis socket and harness that prevented residual limb movements of large amplitude, especially shoulder flexion, abduction, and external rotation. Hence, a large precision error was measured for numerous targets, especially high-located targets like targets 7, 8, and 9 of distance I.

### 4.2. Completion time

The participant achieved the pointing task with a high precision with the prosthetic elbow control in ME-mode. However, the forearm position was not adjusted during the pointing movement itself, but only prior to the movement, making the overall reaching strategy unnatural. The participant's own myoelectric control strategy was particular and adapted to his difficulties to do triceps contractions and co-contractions. If he wanted to change the elbow angle after setting the elbow in the starting position of 90°, he had to do first a co-contraction to fully extend the forearm, then a biceps contraction to flex the elbow and reach the desired position. Therefore, the completion time for movements with ME-mode when the forearm pre-positioning phase was included were increased due to the participant's complex myoelectric control strategy. The participants in the studies of Hussaini et al. (2016) and Metzger et al. (2012) had a similar behavior before starting the actual tasks: the elbow angle of the objects themselves were pre-positioned before the movements such that it was easier to achieve the task. Nonetheless, pre-positioning the prosthesis did not reduce the compensatory behavior, and neither reduced the movement duration.

### 4.3. Compensatory strategies in ME-mode

The pointing strategy chosen by the participant with a myoelectrically-driven elbow, whereby he brought the prosthetic fingertip to the target by leaning the trunk over the table, was the costliest in terms of trunk compensatory movements, as shown in Figure 6. Especially, larger compensatory movements were observed for left-located targets (1, 4, and 7) since the participant's socket prevented external humerus rotation. The analysis of hip anteroposterior motion showed that the participant had an inverted pendulum-type of body behavior whereby ankle dorsiflexion drove the whole body forward, yielding large body inclination angles and trunk displacements. The force distribution analysis showed an important shift toward the left foot during movements with a myoelectrically-driven elbow. The participant's whole body was involved in the movements to compensate for the lack of mobility at the shoulder and elbow joints. Elbow impairment, and even full locking as it is the case of most transhumeral amputees wearing a prosthesis, yields trunk movements of large amplitudes (de Groot et al., 2011; Metzger et al., 2012; Deijs et al., 2016). Metzger et al. (2012) measured trunk displacements of 35 cm in the anteroposterior and mediolateral directions, and shoulder marker cumulative path of 50 cm during reaching movements of transhumeral amputees. Such important modifications of the natural behavior can lead to severe musculoskeletal disorders.

### 4.4. Inter-joint coordination-based control

The results obtained in the present study show that automatic elbow control diminishes trunk compensations. The body inclination were reduced during pointing movements with the prosthetic elbow in A-mode, especially toward targets located at the maximal distance (12.3 ± 5.5° in ME-mode, and 4.5 ± 3.2° in A-mode, averaged over the 9 targets of distance I). Movements with an automatically-driven prosthetic elbow were more natural with synchronous shoulder and elbow motions, as observed in healthy movements. Although the feature was not investigated in this study, the A-mode elbow control strategy enabled simultaneous elbow and end-effector control since residual limb motion drove solely the prosthetic elbow, and myoelectric signals were directed toward wrist and prosthetic hand control. However, residual limb movements were limited by the prosthesis socket and the pain exerted on the stump's extremity due to the prosthesis weight. When compared to able-bodied subjects doing the same movements, the residual limb amplitude was half the amplitude of a healthy arm, as shown in Figure 8. The inter-joint coordination model was implemented on the prosthesis with the assumption that residual limb kinematics were similar to the healthy kinematics included in the generic model training data set. Unfortunately, the residual limb motion assessment demonstrated that it was not the case: important kinematic differences were measured between healthy shoulder movements and residual limb motions. The data sets corresponding to the subjects' shoulder kinematics in Figure 3 were located in different areas of the input data space, and had different shapes. In addition to having the residual limb movement's amplitude reduced by the prosthesis socket and by the harness, the loss of a limb affected the residual limb kinematics by altering the whole sensorimotor loop. The analysis highlighted the fact that residual limb motions and healthy arm motions were significantly different. Also, the weight distribution of a prosthesis is fundamentally different from the one of a healthy limb, especially at the level of the hand and forearm, which generates different dynamical effects such as reaction forces on the prosthesis users' body. The approach tested synthesized two different inter-joint coordinations of able-bodied individuals into one generic coordination model used by the transhumeral amputated participant to control automatically the prosthetic elbow. By combining healthy individuals' data sets, the generic model assimilates the inter-individual variability, but remains different from the prosthesis user's own pointing strategy. Thus, the paradigm whereby the shoulder/elbow coordinations from healthy individuals are driving an elbow prosthesis may not be adapted to prosthesis users, and the presented results justify for the need of a model tailored to the user's residual movements.

### 4.5. Study limitations

The generic model's output depended on the shoulder kinematics, and thus, the prosthetic elbow extended until the residual limb was immobilized. As a result, any adjustment to bring the prosthetic fingertip close to the target after performing the general pointing gesture evoked an elbow extension or flexion, depending on the small residual limb movements. Therefore, before starting the recording, the transhumeral amputated participant was instructed not to adjust the fingertip position once the main residual limb movement was over, which can explain the large precision error. In order to reach the targets with a small error, the participant would have had to know perfectly how to move the residual limb to evoke the adequate prosthetic elbow motion. The A-mode control method of future experiments will include an elbow-locked phase to allow the participant to move the residual limb to adjust the prosthetic end-effector position.

The transhumeral amputated individual that was recruited in the study had received no prior training with automatic elbow control. Before starting the recording, he had 5 min to explore the novel control method. Better results, especially in terms of precision and completion times, could have been expected with training. However, the study was focused on the intuitiveness of the tested control method. More amputated participants will be included in future experiments to investigate the influence of subjects' height and experience with a prosthetic device on the control performance. However, socket designs are a major limitation since they prevent complete residual limb mobility. Also, more gestures will be included in the model to improve its generalization and functionality; the presented automatic control strategy will be tested on functional tasks such as the SHAP test (Wright, 2006; Miller and Swanson, 2009), the clothespin test (Hussaini et al., 2016), or the 400 points assessment test (Gable et al., 1997).

## 5. Conclusion

A transhumeral amputee achieved a pointing task with a prosthetic prototype that included an externally-powered elbow driven by an inter-joint coordination model from healthy individuals' data. The control strategy presented in several studies of the literature was never tested on a device yet. The experiment results showed that the presented approach was beneficial to the prosthesis user as it reduced compensatory movements, and enabled simultaneous control of the elbow (via residual limb motion) and the end-effector (via myoelectric control). Pointing movements became generally more natural when the elbow was automatically-driven by the residual limb. However, the residual limb's amplitudes were limited by the socket and by the pain exerted on the residual limb's extremity. Because of the socket-related impairments and post-amputation sensorimotor modifications, the residual limb movements did not correspond to the expected inputs of the inter-joint coordination model. Therefore, the study illustrates that the utilization of a model of healthy inter-joint coordinations to control prosthetic joints is limited by the residual limb movements that are kinematically different from healthy upper limb movements. It shows the need for novel modeling methods and mapping designs that bring the user back to the center of the control development process in order to achieve a more natural prosthetic motion.

## Author contributions

MM and NJ designed the protocol. MM collected and analyzed the kinematic data of the healthy participants to build the generic model. EdM and NJ built the prosthetic prototype, and EdM collected the data with the amputated participant. AT and NM contacted the amputated participant and organized the experimental session. AT, MM, and NJ participated in the experimental session with the amputated participant. MM, NJ, and AR-B analyzed the data, and wrote the present report.

### Conflict of interest statement

The authors declare that the research was conducted in the absence of any commercial or financial relationships that could be construed as a potential conflict of interest.

## References

[B1] Abboudi R. L. Glass C. Newby N. Flint J. Craelius W. (1999). A biomimetic controller for a multifinger prosthesis. IEEE Trans. Rehabil. Eng. 7, 121–129. 10.1109/86.769401 10391581

[B2] Akhlaghi N. Baker C. Lahlou M. Zafar H. Murthy K. Rangwala H.. (2016). Real-time classification of hand motions using ultrasound imaging of forearm muscles. IEEE Trans. Biomed. Eng. 63, 1687–1698. 10.1109/TBME.2015.2498124 26560865

[B3] Akhtar A. Hargrove L. J. Bretl T. (2012). Prediction of distal arm joint angles from EMG and shoulder orientation for prosthesis control, in Proceedings of the International Conference of the Engineering in Medecine and Biology Society (EMBS) (San Diago, CA), 4160–4163. 10.1109/EMBC.2012.6346883 23366844

[B4] Alshammary N. A. Bennett D. A. Goldfarb M. (2016). Efficacy of coordinating shoulder and elbow motion in a myoelectric transhumeral prosthesis in reaching tasks, in Proceedings of the International Conference on Robotics and Automation (ICRA) (Stockholm), 3723–3728. 10.1109/ICRA.2016.7487559

[B5] Atkins D. J. Heard D. C. Donovan W. H. (1996). Epidemiologic overview of individuals with upper-limb loss and their reported research priorities. J. Prosthet. Orthot. 8, 2–11. 10.1097/00008526-199600810-00003

[B6] Barton J. E. Sorkin J. D. (2014). Design and evaluation of prosthetic shoulder controller. J. Rehabil. Res. Dev. 51, 711–726. 10.1682/JRRD.2013.05.0120 25357185 PMC4578707

[B7] Belter J. T. Segil J. L. Dollar A. M. Weir R. F. (2013). Mechanical design and performance specifications of anthropomorphic prosthetic hands: a review. J. Rehabil. Res. Dev. 50, 599–618. 10.1682/JRRD.2011.10.0188 24013909

[B8] Bernstein N. (1967). The Co-ordination and Regulation of Movements. Oxford: Pergamon Press.

[B9] Biddiss E. Chau T. (2007). Upper-limb prosthetics: critical factors in device abandonment. Am. J. Phys. Med. Rehabil. 86, 977–987. 10.1097/PHM.0b013e3181587f6c 18090439

[B10] Bockemühl T. Troje N. F. Dürr V. (2010). Inter-joint coupling and joint angle synergies of human catching movements. Hum. Mov. Sci. 29, 73–93. 10.1016/j.humov.2009.03.003 19945187

[B11] Bottomley A. (1965). Myo-electric control of powered prostheses. J. Bone Joint Surg. 47, 411–415. 14341052

[B12] Castellini C. Artemiadis P. Wininger M. Ajoudani A. Alimusaj M. Bicchi A.. (2014). Proceedings of the first workshop on peripheral machine interfaces: going beyond traditional surface electromyography. Front. Neurorobot. 8:22. 10.3389/fnbot.2014.00022 25177292 PMC4133701

[B13] Cho E. Chen R. Merhi L.-K. Xiao Z. Pousett B. Menon C. (2016). Force myography to control robotic upper extremity prostheses: a feasibility study. Front. Bioeng. Biotechnol. 4:18. 10.3389/fbioe.2016.00018 27014682 PMC4782664

[B14] Day S. (2002). Important Factors in Surface EMG Measurement. Bortec Biomedical Ltd.

[B15] de Groot J. H. Angulo S. M. Meskers C. G. van der Heijden-Maessen H. C. Arendzen J. H. H. (2011). Reduced elbow mobility affects the flexion or extension domain in activities of daily living. Clin. Biomech. 26, 713–717. 10.1016/j.clinbiomech.2011.03.001 21444133

[B16] Deijs M. Bongers R. Ringeling-van Leusen N. van der Sluis C. (2016). Flexible and static wrist units in upper limb prosthesis users: functionality scores, user satisfaction and compensatory movements. J. Neuroeng. Rehabil. 13, 1–13. 10.1186/s12984-016-0130-0 26979272 PMC4791860

[B17] Farokhzadi M. Maleki A. Fallah A. Rashidi S. (2017). Online estimation of elbow joint angle using upper arm acceleration: a movement partitioning approach. J. Biomed. Phys. Eng. 7, 1–10. 10.22086/jbpe.v0i0.524 29082222 PMC5654137

[B18] Gable C. Xenard J. Makiela E. Chau N. (1997). Evaluation fonctionnelle de la main. Bilan 400 points et tests chiffrés. Ann. Réadaptation Méd. Phys. 40, 95–101. 10.1016/S0168-6054(97)83377-6

[B19] Gibbons D. T. O'riain M. D. Philippe-Auguste S. (1987). An above-elbow prosthesis employing programmed linkages. IEEE Trans. Biomed. Eng. BME-34, 493–498. 10.1109/TBME.1987.325978 3610199

[B20] Hussaini A. Zinck A. Kyberd P. (2016). Categorization of compensatory motions in transradial myoelectric prosthesis users. Prosthet. Orthot. Int. 41, 1–8. 10.1177/0309364616660248 27473642

[B21] Iftime S. D. Egsgaard L. L. Popović M. B. (2005). Automatic determination of synergies by radial basis function artificial neural networks for the control of a neural prosthesis. IEEE Trans. Neural Syst. Rehabil. Eng. 13, 482–489. 10.1109/TNSRE.2005.858458 16425830

[B22] Kaliki R. R. Davoodi R. Loeb G. E. (2008). Prediction of distal arm posture in 3-D space from shoulder movements for control of upper limb prostheses. Proc. IEEE 96, 1217–1225. 10.1109/JPROC.2008.922591

[B23] Lacquaniti F. Soechting J. F. (1982). Coordination of arm and wrist motion during a reaching task. J. Neurosci. 2, 399–408. 7069463 10.1523/JNEUROSCI.02-04-00399.1982 PMC6564249

[B24] Latash M. L. Aruin A. S. Zatsiorsky V. M. (1999). The basis of a simple synergy: reconstruction of joint equilibrium trajectories during unrestrained arm movements. Hum. Mov. Sci. 18, 3–30. 10.1016/S0167-9457(98)00029-3

[B25] Lipschutz R. D. Lock B. Sensinger J. Schultz A. E. Kuiken T. A. (2011). Use of a two-axis joystick for control of externally powered, shoulder disarticulation prostheses. J. Rehabil. Res. Dev. 48, 661–668. 10.1682/JRRD.2010.04.0072 21938653 PMC4313786

[B26] Madgwick S. O. (2010). An Efficient Orientation Filter for Inertial and Inertial/Magnetic Sensor Arrays. Report x-io and University of Bristol.

[B27] Merad M. de Montalivet É. Roby-Brami A. Jarrassé N. (2016a). Intuitive prosthetic control using upper limb inter-joint coordinations and IMU-based shoulder angles measurement: a pilot study, in Proceedings of the International Conference on Intelligent Robots and Systems (Daejeon), 5677–5682.

[B28] Merad M. Roby-Brami A. Jarrassé N. (2016b). Towards the implementation of natural prosthetic elbow motion using upper limb joint coordination, in Proceedings of the International Conference on Biomedical Robotics and Biomechatronics (Singapore), 829–834.

[B29] Metzger A. J. Dromerick A. W. Holley R. J. Lum P. S. (2012). Characterization of compensatory trunk movements during prosthetic upper limb reaching tasks. Arch. Phys. Med. Rehabil. 93, 2029–2034. 10.1016/j.apmr.2012.03.011 22449551

[B30] Micera S. Carpaneto J. Posteraro F. Cenciotti L. Popovic M. Dario P. (2005). Characterization of upper arm synergies during reaching tasks in able-bodied and hemiparetic subjects. Clin. Biomech. 20, 939–946. 10.1016/j.clinbiomech.2005.06.004 16061318

[B31] Mijovic B. Popovic M. Popovic D. B. (2008). Synergistic control of forearm based on accelerometer data and artificial neural networks. Braz. J. Med. Biol. Res. 41, 389–397. 10.1590/S0100-879X2008005000019 18516468

[B32] Miller L. A. Swanson S. (2009). Summary and recommendations of the academy's state of the science conference on upper limb prosthetic outcome measures. J. Prosthet. Orthot. 21, P83–P89. 10.1097/JPO.0b013e3181ae974d

[B33] Montagnani F. Controzzi M. Cipriani C. (2015). Exploiting arm posture synergies in activities of daily living to control the wrist rotation in upper limb prostheses: a feasibility study, in EMBC (Milan), 2462–2465. 10.1109/EMBC.2015.7318892 26736792

[B34] Østlie K. Franklin R. J. Skjeldal O. H. Skrondal A. Magnus P. (2011). Musculoskeletal pain and overuse syndromes in adult acquired major upper-limb amputees. Arch. Phys. Med. Rehabil. 92, 1967–1973. 10.1016/j.apmr.2011.06.026 22133243

[B35] Roby-Brami A. Bennis N. Mokhtari M. Baraduc P. (2000). Hand orientation for grasping depends on the direction of the reaching movement. Brain Res. 869, 121–129. 10.1016/S0006-8993(00)02378-7 10865066

[B36] Sierra González D. Castellini C. (2013). A realistic implementation of ultrasound imaging as a human-machine interface for upper-limb amputees. Front. Neurorobot. 7:17. 10.3389/fnbot.2013.00017 24155719 PMC3804922

[B37] Silva J. Chau T. (2003). Coupled microphone-accelerometer sensor pair for dynamic noise reduction in MMG signal recording. Electron. Lett. 39, 1–2. 10.1049/el:20031003

[B38] Stulp F. Sigaud O. (2015). Many regression algorithms, one unified model: a review. Neural Netw. 69, 60–79. 10.1016/j.neunet.2015.05.005 26087306

[B39] Wright F. V. (2006). Measurement of functional outcome with individuals who use upper extremity prosthetic devices: current and future directions. J. Prosthet. Orthot. 18, 46–56. 10.1097/00008526-200604000-00006

[B40] Wright T. W. Hagen A. D. Wood M. B. (1995). Prosthetic usage in major upper extremity amputations. J. Hand Surgery 20, 619–622. 10.1016/S0363-5023(05)80278-3 7594289

